# Refractometer assessment of colostral and serum IgG and milk total solids concentrations in dairy cattle

**DOI:** 10.1186/s12917-014-0178-7

**Published:** 2014-08-15

**Authors:** Munashe Chigerwe, Jill V Hagey

**Affiliations:** 1 Department of Medicine and Epidemiology, University of California-Davis, School of Veterinary Medicine, One Shields Avenue, Davis 95616, CA, USA

**Keywords:** Colostrum, Milk, Serum, Immunoglobulin, Milk total solids

## Abstract

**Background:**

Estimation of the quantity of colostral IgG or serum IgG absorbed following ingestion of colostrum by calves is essential for monitoring the effectiveness of colostrum feeding practices on dairy farms. Milk total solids concentrations determination is a critical part of quality assessment of nonsaleable whole milk prior to feeding to calves. To date, on-farm methods to assess colostral IgG, serum IgG or milk total solids concentrations have been performed separately with various instruments. The objective of this study was to evaluate the diagnostic performance of a single electronic, hand-held refractometer for assessing colostral and serum IgG concentrations and milk total solids in dairy cattle. Colostral IgG, serum IgG and milk total solids concentrations were determined by the refractometer. Corresponding analysis of colostral and serum IgG concentrations were determined by radial immunodiffusion (RID) while milk total solids were determined by spectrophotometry. Sensitivity and specificity of the refractometer for colostrum and serum samples were calculated as determined by RID. Sensitivity and specificity of the refractometer for milk samples was calculated as determined by spectrophotometry.

**Results:**

The sensitivity of the refractometer was 1 for colostral IgG, serum IgG and milk total solids determinations. Specificity of the refractometer was 0.66, 0.24 and 0 for colostral IgG, serum IgG and milk total solids determinations, respectively. The refractometer underestimated colostral IgG, serum IgG and milk total solids concentrations compared to the concentrations determined by RID or spectrophotometry.

**Conclusions:**

The refractometer was an acceptable, rapid, convenient on-farm method for determining colostral IgG and milk total solids. The refractometer was not an acceptable method for determination of serum IgG concentrations as it severely underestimated the serum IgG concentrations.

## Background

Estimation of IgG concentration in colostrum prior to feeding to calves is a useful tool to improve calf health by ensuring adequate transfer of colostral immunoglobulins. Several on-farm methods for estimating colostral IgG concentrations have been reported including hydrometers [1],[2], weight of first milking colostrum [3], immunoassay [4], and electronic refractometer [5] with variable test sensitivity and levels of practical application. Estimation of the quantity of IgG absorbed following ingestion of colostrum by calves is essential for monitoring the effectiveness of colostrum feeding practices on dairy farms. Practical methods used for estimating serum IgG following ingestion of colostrum in dairy calves include serum total protein determination by refractometry [6],[7], sodium sulfite [8], and turbidimetric methods [9]. Radial immunodiffusion (RID) is considered the reference method for determination of colostral or serum IgG concentrations and is therefore utilized in diagnostic and laboratory settings [10].

Nonsaleable milk includes postparturient milk collected after the first-milking colostrum for the first few days of a cow’s lactation as well as discarded milk from lactating cows undergoing treatment for various disease conditions. Nonsaleable milk can be fed neonatal dairy calves following pasteurization. Milk total solids (sum of percentage fat and solids non-fat) assessment is critical in the determination of the nutritional value of the nonsaleable milk prior to feeding to calves because the total solids concentrations are variable. In instances where the total solids concentrations are insufficient, additional ingredients such as milk replacer may be added to the milk to achieve target total solids concentrations. Practical methods used to assess whole milk total solids in dairy milk include refractometry [11],[12] while spectrophotometry is considered the reference method for evaluating milk total solids [12].

Previous refractometers estimated colostral IgG or total solids by reporting a Brix value (measure of refractive index), which is then correlated to colostral IgG or total solid concentrations. Additionally, previous practical methods to estimate colostral IgG concentration, serum IgG concentrations in calves or total solids in milk were performed separately with different instruments. A more practical approach would be utilization of a single on-farm instrument that quantitatively reports colostral IgG, serum IgG and total solids without the need to correlate Brix test results with colostral or serum IgG concentrations or total solids. To the authors’ knowledge, no studies have evaluated the use of a single instrument to quantitatively estimate colostral or serum IgG concentrations and milk total solids. The purpose of this study was to evaluate the practical diagnostic utility of a single on-farm refractometer capable of estimating colostral or serum IgG concentrations and milk total solids with test results reported in g/L for colostrum and serum and % total solids for milk.

## Methods

### Samples size calculation and sample collection

Colostral sample size calculation using previously reported methods [13] were based on reported sensitivity of 0.75 [5] of the electronic refractometer in assessing colostral quality. Serum sample size determination using previously reported methods [13] was based on the sensitivity of 0.89 [7] of refractometers for assessing passive transfer status in serum of calves. Sample size calculation using standard methods [14] for the required milk samples was based on the previously reported correlation coefficient of 0.93 between the refractometer and spectrophotometry results [12]. In all sample size calculations a power of ≥ 0.80 and alpha = 0.05 was considered. A minimum sample size of 43 colostral, 39 serum and 54 milk samples were required.

To increase the variability in colostral or serum IgG concentrations, samples were collected from more than one farm and or from 2 dairy breeds. Fresh, pooled colostrum samples from 3 Jersey dairy farms were collected. Blood samples were collected from 2-day old heifer calves from 2 dairy farms (1 Jersey farm and 1 Holstein farm) followed by serum harvesting by centrifugation within 2 hours after collection. The colostral and serum samples were stored at -20°C until IgG concentration was measured using a hand-held refractometer (Palm Abbe PA203x, Misco, Cleveland, Ohio, USA) and single RID. According to the manufacturer, the refractometer analysis can be performed on non-frozen colostrum or serum as well as colostrum or serum that has been frozen only once. Analysis of colostrum and serum was performed within 8 weeks after collection. Milk samples were collected from a single dairy farm raising both Holsteins and Jersey cows. Total solids were determined by the refractometer and spectrophotometry on non-frozen fresh milk samples. All sample collection and analysis were conducted between June 2013 and October 2103. Permission to collect study samples was granted by the participating dairy farms. The University of California, Davis Animal Care and Use Committee approved the study.

### Sample analysis

Stored colostrum and samples were thawed at room temperature (20-24 C) while milk total solids was determined on fresh non-pasteurized milk samples. The scales for milk solids, colostral or serum IgG on the refractometer were automatically temperature compensated for aqueous sucrose solutions at 20°C between the temperatures of 10 and 40°C. An aliquot (0.4 ml) of deionized water was placed on the prism well of the refractometer to obtain a standardized reading as per manufacturer’s recommendations. Following removal of the deionized water, the refractometer was set to determine colostral or serum IgG or milk total solids. An aliquot (0.4 ml) of the samples was placed in the prism well of the refractometer and result displayed within a minute. The refractometer reported colostral IgG in g/L, serum IgG in g/L and milk total solids in %. Range of measurement for colostral IgG, serum IgG and milk total solids were 22-82 g/L, 2-25 g/L and 5-15%, respectively. In order to ensure a wide range of milk total solids, 9 milk samples were diluted 1:2 with Tris buffered saline.

Single RID using a commercial kit (Bovine RID Kit, Triple J Farms, Bellingham, Washington, USA) was performed on the same colostral and serum samples evaluated by the refractometer. Briefly, RID plates containing specific anti-bovine IgG, agarose gel, 0.1 M phosphate buffer pH 7.0, 0.1% sodium azide as a bacteriostatic agent and 1 μg/ml amphotericin B as an antifungal agent stored in a refrigerator at 4°C were warmed at room temperature (20-24 C). An aliquot (5 μl) of the provided reference serum at 3 different concentrations, were pipetted into the first 3 RID wells. An aliquot (5 μl) of serum (diluted 1:2 with phosphate buffered saline) or colostrum samples (diluted 1:4 with phosphate buffered saline) were pipetted into the remaining individual RID wells. The plates were incubated at room temperature (20-24°C) for 24 hours. The diameters of the zones of precipitation were measured using a digital RID plate reader (RID plate reader, The Binding Site Inc, San Diego, California, USA) after 24 hours. Colostral or serum sample IgG concentrations were determined by comparing the diameter of the zones of precipitation with a standard curve generated by the reference serum. The regression equation generated in this manner (R^2^ = 0.97-0.99) accurately predicted inoculum IgG concentration. The reference serum was included in all plates to minimize plate-to-plate variations. Minimum detectable IgG concentration was 196 mg/dL using the RID. Milk samples evaluated by the refractometer were analyzed for total solids at a certified dairy analytical laboratory (Sierra Dairy Labs, Tulare, California, USA) by a mid-infrared method [15] using a spectrophotometer (Bently 150, Bently Instruments, Chaska, Minnesota, USA).

### Data analysis

Normality of data was checked using the Shapiro-Wilk test. When data was normally distributed the mean was reported as the measure of central tendency. For all statistical tests single RID was considered the reference method for determining colostral and serum IgG concentrations, while spectrophotometry was considered the reference method for determining milk total solids. Descriptive statistics for colostral or serum IgG concentrations and milk total solids were performed. Samples with colostral IgG < 22 or > 82 g/L, serum IgG concentrations < 2 or > 25 g/L as determined by the RID were excluded from the study because of the detection limit of the refractometer. Likewise milk samples with total solids < 5 or > 15% as determined by spectrophotometer were excluded from the study.

Diagnostic sensitivity and specificity (95% CI) of the refractometer was calculated based on the test results using 2 × 2 frequency tables [13]. A concentration of < 50 g/L of IgG was considered the cut-off point for indicating colostral samples with insufficient IgG [1],[5]. A cut off-off point of < 10 g/L was considered indicative of inadequate colostral IgG transfer in calf serum [16],[17]. A cut-off point of < 12% was considered indicative of insufficient total solids in whole milk [12]. Sensitivity of the refractometer was defined as the probability of a test result indicating an inadequate colostral (<50 g/L) or serum (<10 g/L) IgG concentrations as determined by means of RID. Specificity of the refractometer was defined as the probability of a test result indicative of an adequate colostral (≥50 g/L) or serum (≥10 g/L) IgG concentrations as determined by means of RID. Likewise, for milk, the sensitivity of the refractometer was defined as the probability of a test result indicative of inadequate milk total solids (<12%) as determined by spectrophotometry. Specificity of the refractometer was defined as the probability of a test result indicative of adequate milk total solids (≥12%) as determined by spectrophotometry. As a result of differences in prevalence of colostral or serum samples with inadequate IgG concentrations or milk samples with inadequate milk total solids, likelihood ratios (prevalence independent) for a positive or negative test were determined instead of predictive values (prevalence dependent) for a positive or negative test. Likelihood ratios were calculated using standard methods as previously described [13].

In order to assess the ability of the refractometer to provide accurate measurements as indicated by how closely the test results matched with RID or spectrophotometry, precision of the refractometer was determined using the Bland-Altman (limits of agreement plots) method [18]. For each sample and the measured analyte (colostral IgG, serum IgG or milk total solids) the bias was calculated as the concentration determined by RID or spectrophotometer minus the corresponding paired analyte concentration as determined by the refractometer. Mean bias and 95% (mean ± 1.96 SD) limits of agreement were calculated. A positive bias estimation indicated that the refractometer underestimated the concentration of the analyte, whereas a negative bias estimate indicated that the refractometer overestimated the concentration of the analyte compared with concentration of the same analyte as determined by RID or spectrophotometer. Where applicable, data analysis was performed using a commercial statistical software (Prism 6, GraphPad Inc, La Jolla, California, USA). Values of *P* < 0.05 were considered significant.

## Results

Sixty-four colostral, 46 serum and 74 milk samples were collected. Mean ± SD for colostral and serum IgG concentrations were 61.0 ± 11.1 g/L and 18.5 ± 8.6 g/L, respectively, as determined by RID. Mean ± SD for milk total solids was 10.2 ± 1.7%. Proportion of samples with colostral IgG < 50 g/L was 17.2% (11/64) and proportion of samples with serum IgG < 10 g/L was 17.4% (8/46) as determined by RID. Proportion of samples with milk total solids < 12% was 87.8% (65/74) as determined by spectrophotometry.

### Colostral IgG

**Figure 1:**
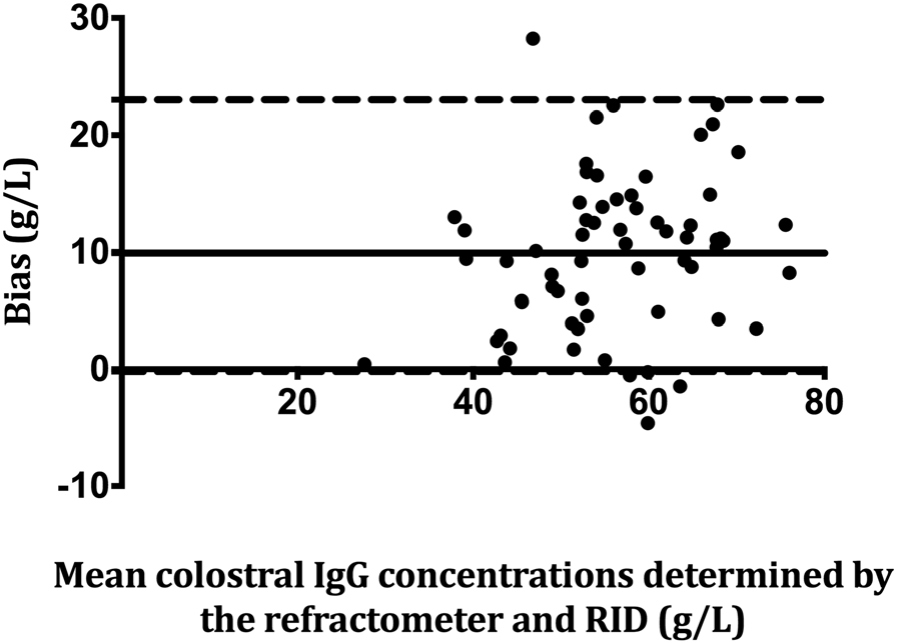
**Bland-Altman plots of colostral IgG concentrations concurrently determined by RID and refractometer.** Bias was calculated as the colostral IgG concentration determined by RID minus the corresponding paired analyte concentration as determined by the refractometer. The solid represents the mean bias whereas the dashed lines represent the 95% limits of agreement (mean ± 1.96 SD).

Sensitivity and specificity of the refractometer for detecting colostral samples with < 50 g/L was 1 (95% CI, 1) and 0.66 (95% CI, 0.53-0.79) respectively. Likelihood ratio of a positive test was 2.9 (95% CI, 1). Likelihood ratio for a negative test could not be calculated because there were no false negatives, resulting in a denominator of zero. Mean bias based on the Bland-Altman plots was 9.9 g/L (95% limits of agreement, -3.1-23.0) indicating that the refractometer underestimated sample colostral IgG concentrations by 9.9 g/L, on average. The Bland-Altman plot for colostral IgG determination is represented in Figure 1.

### Serum IgG concentrations

**Figure 2:**
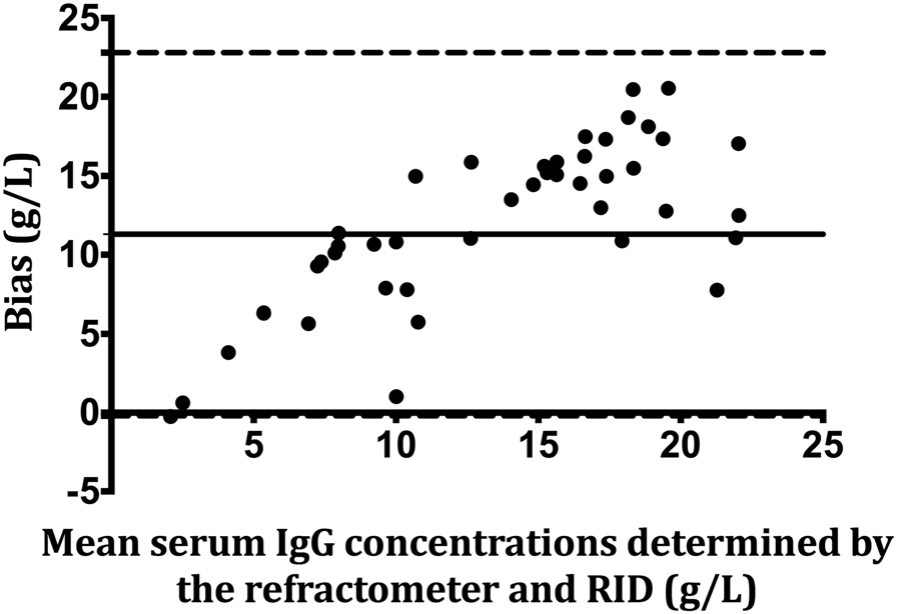
**Bland-Altman plots of serum IgG concentrations concurrently determined by RID and refractometer.** Bias was calculated as the serum IgG concentration determined by RID minus the corresponding paired analyte concentration as determined by the refractometer. The solid represents the mean bias whereas the dashed lines represent the 95% limits of agreement (mean ± 1.96 SD).

Sensitivity and specificity of the refractometer for detecting serum samples with IgG < 10 g/L was 1 (95% CI, 1) and 0.24 (95% CI, 0.1-0.38) respectively, as determined by RID. Likelihood ratio of positive test was 1.3 (95% CI, 1). Likelihood ratio for a negative test could not be calculated because there were no false negatives, resulting in a denominator of zero. Mean bias based on the Bland-Altman plots was 11.3 g/L (95% limits of agreement, -0.2-22.8) indicating that the refractometer underestimated sample serum IgG concentrations by 11.3 g/L, on average. The Bland-Altman plot for serum IgG determination is represented in Figure 2.

### Milk total solids

**Figure 3:**
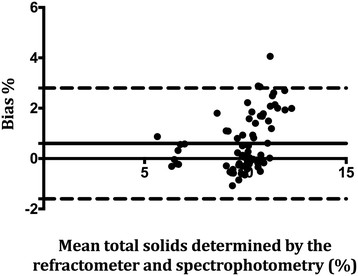
**Bland-Altman plots of milk total solids concentrations concurrently determined by spectrophotometry and refractometer.** Bias was calculated as the total solids concentration determined by spectrophotometry minus the corresponding paired analyte concentration as determined by the refractometer. The solid represents the mean bias whereas the dashed lines represent the 95% limits of agreement (mean ± 1.96 SD).

Sensitivity and specificity for detecting samples with milk total solids < 12% was 1 (95% CI, 1) and 0 (95% CI, 0) respectively, as determined by spectrophotometry. Likelihood ratio for a positive test was 1 (95% CI, 1). Likelihood ration for a negative test was not determined because there were no true negatives resulting in a denominator of zero. Mean bias based on the Bland-Altman plots was 0.6 (95% limits of agreement, -1.6-2.8) indicating that the refractometer underestimated milk total solids by 0.6% on average. The Bland-Altman plot for milk total solids is represented in Figure 3.

## Discussion

The main findings from the present study indicated that the refractometer maybe beneficial for the rapid assessment of colostral IgG and milk total solids but should be used with caution when evaluating serum IgG concentrations in calves. The main advantage of the refractometer over the previous methods for assessing colostral IgG, serum IgG or milk total solids is the potential convenience of a single instrument that assess all 3 analytes. The refractometer had a sensitivity of 1 for identifying samples with low colostral IgG, serum IgG or milk total solids concentrations. However, the high sensitivity with a low specificity results in misclassifications of samples with adequate colostral IgG, serum IgG and milk total solids as inadequate. An effective screening test should have high sensitivity to ensure high predictive value for a negative test and ensure identification of all samples with low colostral IgG, serum IgG or milk total solids.

The refractometer measures the refractive index. Some refractometers are designed to report the test result as a refractive index [12],[19] where as others report the test result as % total solids, as in this study. The refractive index can be used to estimate total solids in cases where the refractometer reports a refractive index. The refractive index varies with the concentration of the compound, temperature of the compound and the wavelength of the light. The refractive index for solutions or transparent liquids with particles sizes between 0.1-0.5 μm in diameter is given by the additive refractive index from each particle [19]. The refractive index of colostrum or milk is considered difficult to measure because of the presence of fat globules, casein micelles and other non-uniform sized globules [20].

Assessment of colostral IgG concentrations is important to determine the colostral volume required to be fed to a calf or storage, assuming that 150-200 g total IgG is required for adequate transfer of colostral immunoglobulilns [21]. The refractometer had a higher sensitivity compared to other quantitative methods for assessing colostral IgG that includes the hydrometer [2],[5], refractometer [5] and immunoassay [4]. The advantage of the refractometer (colostral temperature range for determination, 10-40°C) over the hydrometer (colostral temperature range for determination, 22-24°C) is that fresh colostral (mean temperature, 34.6°C) [5] IgG concentration determination does not require cooling. The advantage of the test results of the refractometer reported in this study compared to the previous evaluation of another refractometer [5] is that the reported units are in g/L compared to Brix units. Colostral IgG reported in g/L are easier to interpret when determining colostral volume to be fed to the calf compared to Brix units. While another advantage of the refractometer in this study was that it reported a range (22-82 g/L) of colostral IgG concentration, the immunoassay [4] results were only qualitative (<50 g/L or > 50 g/L). It should be noted that the refractometer underestimated the colostral IgG concentration by approximately 10 g/L as indicated by the mean bias for the refractometer being 9.9 g/L. The implications of this underestimation of colostral IgG concentrations is likely to be clinically insignificant as it may only result in increase in volume of colostrum fed to the calf.

Evaluation of passive transfer status in calves is important for monitoring colostrum feeding practices on dairy farms or as part of a treatment management plan for calves with failure of passive immunity in clinic settings. The sensitivity of 1, of the refractometer for identifying serum samples with IgG concentrations < 10 g/L was higher compared to other common methods used to assess passive transfer status that include serum total protein and sodium sulfite [6],[8],[22]. The advantage of the refractometer over other practical methods is that it reports serum IgG concentration. However, the mean bias for the refractometer is 11.3 g/L indicating that the refractometer severely underestimated the serum IgG concentrations by 11.3 g/L on average, compared with the RID. Considering that serum IgG concentrations of ≥ 10 g/L are considered indicative of adequate transfer of colostral immunity [16],[17], as an example a serum sample result of 5 g/L (indicative failure of passive transfer of colostral immunity) using this particular digital electronic refractometer may have a true IgG concentration of 16.3 g/L (5 + 11.3), which is indicative of adequate transfer of colostral immunoglobulins. Thus, practically, using results from this particular refractometer alone for estimating serum IgG concentrations may unnecessarily change colostrum feeding practices on dairy farms or implement inappropriate treatment plan for calves in clinic settings. Therefore, we concluded that the refractometer assessed in study was not an acceptable method for determination of serum IgG concentrations.

Calves fed pasteurized milk had improved weight gain and reduced morbidity and mortality compared to calves fed milk replacer [23],[24]. However the nutrient value of milk with low total solids (<13%) can be improved by adding milk replacer following assessment of milk total solids. Mean total solids from the samples reported in this study were lower than recommended of levels of 12.5-13% [11],[12]. The sensitivity of the refractometer in detecting milk samples with < 12% total solids was perfect. The mean bias of the refractometer (0.6%) indicated that the refractometer underestimated the milk total solids by 0.6%, on average. Based on these results, the refractometer is a recommended method for assessing milk total solids because the underestimation is of smaller magnitude, on average.

Although the colostral IgG concentrations in this study were variable, it is important to note that only samples from Jersey cows were evaluated. While other studies indicated that colostral IgG concentrations were not different among five dairy breeds (Holsteins, Jersey, Guernsey, Brown Swiss and Aryshire) [25], other studies reported significantly higher colostral IgG concentrations in Guernsey compared to Holsteins under the same management conditions [26]. However considerable within breed variations in colostral IgG concentrations has been reported in Holsteins [3],[27],[28] and Jersey cows [29],[30]. Thus, it is mostly likely that the variability in colostral samples in this study from 3 different farms were sufficient to evaluate the refractometer. It should be noted that the refractometer was assessed in one geographical area, thus the external validity of its performance may vary. Thus it is recommended to individually validate the refractometer in different geographical conditions where colostral IgG, serum IgG or milk solids concentration are different.

## Conclusions

Results of the present study indicated that the use the single refractometer is an acceptable on farm method for quantitatively evaluating colostral IgG concentrations and milk total solids in dairy cattle. The refractometer was not an acceptable method for determination of serum IgG concentrations in calves. The refractometer can be used by producers to assess colostral IgG and milk total solids concentrations prior to feeding calves.

## Competing interests

The authors declare that they have no competing interests.

## Authors’ contributions

MC conceived the experiment, designed the experiment, performed statistical analysis and wrote the manuscript. JV performed sample analysis and proof read the manuscript. Both authors read and approved the final manuscript.
